# Supplementary material for: YidC from Escherichia coli Forms an Ion-Conducting Pore upon Activation by Ribosomes
Source: Biomolecules. 2023 Dec 11;13(12):1774. doi: 10.3390/biom13121774 (PMC10741985; doi:10.3390/biom13121774)
Supplement: Supplementary file 1 [file biomolecules-13-01774-s001.zip › biomolecules-2729583-supplementary.pdf]

Supplemental Information

YidC from *Escherichia coli* Forms an Ion-Conducting Pore upon Activation by Ribosomes

Denis G. Knyazev [1,†], Lukas Winter [1,†], Andreas Vogt [2,3,4], Sandra Posch [1], Yavuz Öztürk [2], Christine Siligan [1], Nikolaus Goessweiner-Mohr [1], Nora Hagleitner-Ertugrul [1], Hans-Georg Koch [2,3] and Peter Pohl [1,*]

The Supplement consists of five figures and the coordinates of the AlphaFold model (pdb format).

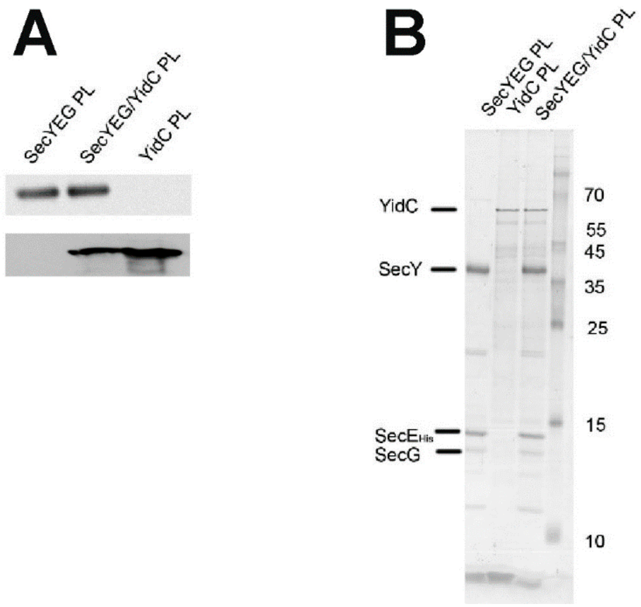

Figure S1. Purity of reconstituted samples. A: Western blotting demonstrates that purified YidC was not contaminated with SecYEG. The indicated proteoliposomes (25 pmol protein) were separated on SDS-PAGE and after western blotting, the membrane was cut into two pieces. The lower part was decorated with antibodies against YidC and the upper part with antibodies against SecY.. B: Coomassie-stain of proteoliposomes with reconstituted SecYEG, YidC or both.

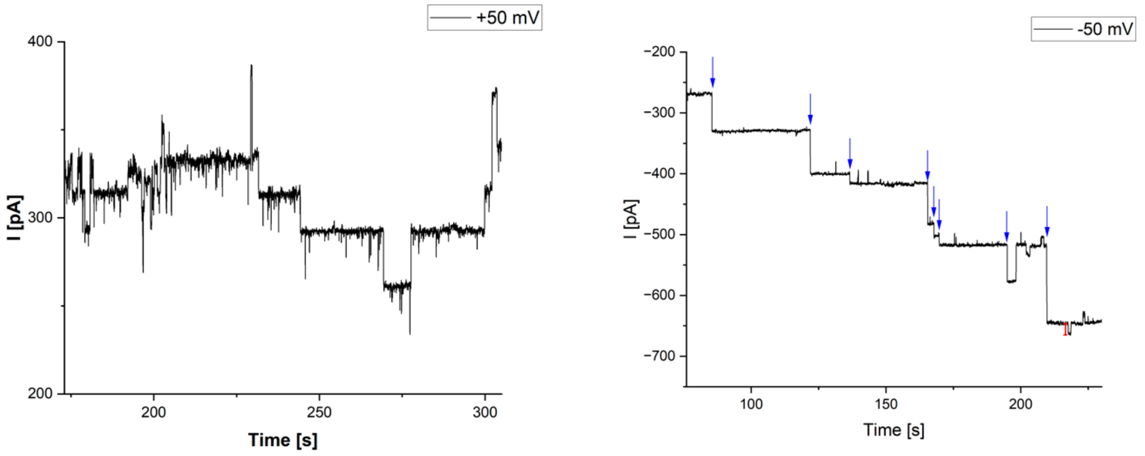

Figure S2. Multiple YidC channels activated by ribosomes. Left: the lipid membrane contained about 10 open YidC channels. Right: fusion events of YidC-proteoliposomes with the planar membrane. The red bar marks the amplitude corresponding to a single YidC channel. At the end of the record, the planar membrane contained more than 20 open YidC channels.

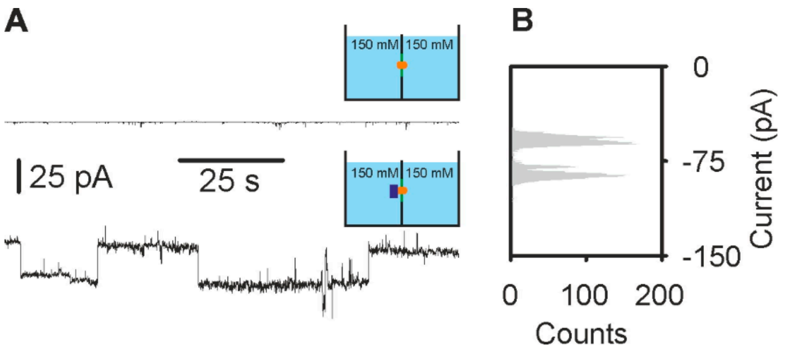

Figure S3. **YidC exhibits channel activity only after ribosome binding. (A)** Traces of current through lipid bilayer containing YidC. The bilayer was folded from monolayers formed on top of a proteoliposome suspension (see *Methods*) in symmetric salt conditions: 150 mM KCl and 50 mM K-HEPES, pH 7.5 in both compartments. Upper trace: negative control. Zero current through the lipid bilayer, containing YidC, before the addition of empty ribosomes. The same was observed when only ribosomes were added to the bare bilayer (data not shown). Lower trace: current through the lipid bilayer showing single channel activity after addition of empty ribosomes. -75 mV were applied across the bilayer. **(B)** Current histogram corresponding to the lower trace in (A). The distance between the two neighbor peaks on the histogram corresponds to the current through a single channel, I_{SC} = -24,7 ± 4 pA or single channel conductivity g = 329 ± 53 pS. Colored schemes show the chamber with two compartments filled with the solution of the indicated ionic strength. The compartments are separated by the lipid bilayer (green), containing YidC (orange) and bound ribosome (purple).

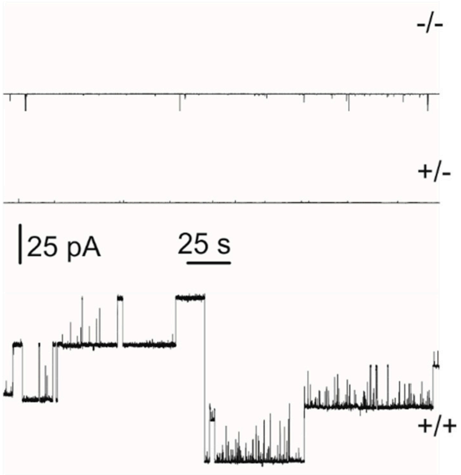

Figure S4. Signal peptide is not able to open YidC channels. Single channel traces shown were recorded at a transmembrane salt gradient of 450: 150 mM KCl and a transmembrane potential of -50 mV. Both compartments were buffered (pH 7.5) by 50 mM K-HEPES. The upper panel shows no channel activity in the absence of both, the signal peptide of cytochrome O oxidase's A subunit and the ribosome (-/-). Upon addition of solely signal peptide (+/-), no channel activity could be observed. The further addition of ribosomes (+/+) resulted in channel activity.

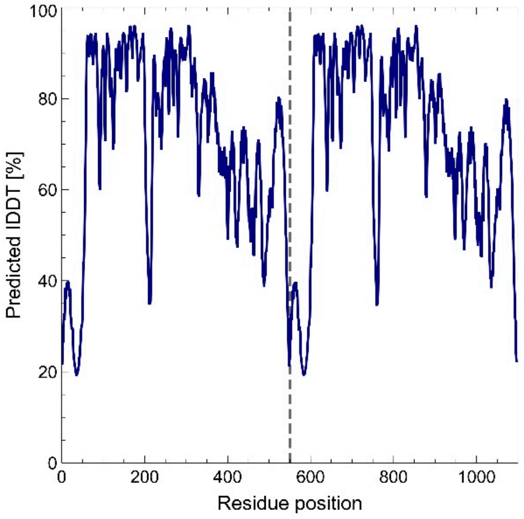

Figure S5. Quality assessment of the dimer model generated by AlphaFold. Using the lDDT – the local distance difference test value [%] - to measure model confidence reveals an overall high model confidence. The only exceptions are exposed loop or transition areas and the termini of the YidC monomers.

:
